# Genetic Dissection of the *Ity3* Locus Identifies a Role for *Ncf2* Co-Expression Modules and Suggests *Selp* as a Candidate Gene Underlying the *Ity3.2* Locus

**DOI:** 10.3389/fimmu.2014.00375

**Published:** 2014-08-12

**Authors:** Rabia Tahir Khan, Marie Chevenon, Kyoko E. Yuki, Danielle Malo

**Affiliations:** ^1^Department of Human Genetics, McGill University, Montreal, QC, Canada; ^2^Complex Traits Group, McGill University, Montreal, QC, Canada; ^3^Department of Medicine, McGill University, Montreal, QC, Canada

**Keywords:** *Salmonella*, *Ity3*, innate immunity, murine model for typhoid, selectin P, *Ncf2*

## Abstract

Typhoid fever and salmonellosis, which are caused by *Salmonella typhi* and *typhimurium*, respectively, are responsible for significant morbidity and mortality in both developed and developing countries. We model typhoid fever using mice infected with *Salmonella typhimurium*, which results in a systemic disease, whereby the outcome of infection is variable in different inbred strains of mice. This model recapitulates several clinical aspects of the human disease and allows the study of the host response to *Salmonella typhimurium* infection *in vivo*. Previous work in our laboratory has identified three loci (*Ity, Ity2*, and *Ity3*) in the wild-derived MOLF/Ei mice influencing survival after infection with *Salmonella typhimurium*. Fine mapping of the *Ity3* locus indicated that two sub-loci contribute collectively to the susceptibility of B6.MOLF-*Ity/Ity3* congenic mice to *Salmonella* infection. In the current paper, we provided further evidence supporting a role for *Ncf2* (neutrophil cytosolic factor 2 a subunit of NADPH oxidase) as the gene underlying the *Ity3.1* sub-locus. Gene expression profiling indicated that the *Ity3.1* sub-locus defined a global gene expression signature with networks articulated around *Ncf2*. Furthermore, based on differential expression and complementation analysis using *Selp* (selectin-P) knock-out mice, *Selp* was identified as a strong candidate gene for the *Ity3.2* sub-locus.

## Introduction

*Salmonella enterica*, an intracellular Gram-negative bacterium, is the causative agent for a wide spectrum of clinical diseases with manifestations ranging from asymptomatic carriers, self-limiting gastroenteritis to fatal systemic infection (1, 2). There are over 2500 *Salmonella* serovars, of which, some are host adapted such as serovar Typhi and Paratyphi in humans, while others, such as Typhimurium and Enteritidis, have a broad host range and are capable of infecting multiple organisms. In humans, *Salmonella typhi* causes a systemic disease, typhoid fever, which has a global health burden of 26.9 million cases and 200,000 deaths annually (3). In humans, *Salmonella typhimurium* is the causative agent of salmonellosis, a self-limiting gastroenteritis that results from the consumption of contaminated food or water. The emergence of multi-drug resistant strains of *Salmonella* in recent years highlights the need for a more comprehensive understanding of the pathogenesis of *Salmonella* infection and for the identification of novel drug targets for vaccines and therapeutics (4).

*Salmonella* is a natural pathogen of mice and infection with *Salmonella typhimurium* results in a typhoid-like systemic disease. This murine experimental model has been used to identify several genes and pathways involved in disease pathogenesis (5–9). As there is limited genetic variation within the classical inbred strains, the use of wild-derived strains of mice, such as MOLF/Ei contributes added genetic diversity and has allowed for the identification of novel genes that play an important role in innate immunity (10–13). Classical and wild-derived strains of mice exhibit a range of susceptibilities to *Salmonella* infection; for example, the C57BL/6J classical inbred strain are extremely susceptible to infection with *Salmonella typhimurium* due to a mutation in *Slc11a1* (solute carrier family 11 member 1), while the 129 sub-strains are highly resistant (14). The wild-derived mouse strain, MOLF/Ei is also susceptible to infection despite carrying functional copies of genes known to be important in *Salmonella* infection, such as *Slc11a1* and *Tlr4* (toll-like receptor 4) (6, 10).

In order to identify the genetic determinants involved in the susceptibility of MOLF/Ei mice to *Salmonella* infection, we have previously used linkage analysis in an F2 panel of (C57BL/6 × MOLF/Ei) mice to identify two loci linked to host defense against *Salmonella typhimurium, Ity2* (Immunity to Typhimurium locus 2) and *Ity3* (10, 12). The MOLF/Ei allele at the *Ity2* locus improves resistance to infection, whereas MOLF/Ei allele at the *Ity3* locus confers susceptibility (15). Validation and fine mapping of *Ity3* locus were done using congenic B6.MOLF-*Ity/Ity3* mice (12) and a panel of 12 sub-congenic mice (16). Using this approach, the *Ity3* locus was refined to a 24 Mb interval and was shown to carry two sub-loci, *Ity3.1* and *Ity3.2* that together contribute to increased susceptibility to infection (16). The *Ity3.1* sub-locus controls NADPH oxidase activity and is characterized by decreased reactive oxygen species (ROS) production, reduced inflammatory cytokine response, and increased bacterial burden. The *Ity3.2* sub-locus is characterized by a hyper-responsive inflammatory cytokine phenotype after exposure to *Salmonella* (16). Sequencing, expression, and functional data support the candidacy of *Ncf2* (neutrophil cytosolic factor 2 a subunit of NADPH oxidase) as the gene underlying the *Ity3.1* sub-locus (13).

In the current study, we used global expression profiling to better understand the genetic networks that are being influenced by the *Ity3* sub-loci and to identify potential candidate genes for the *Ity3.2* sub-locus. We illustrate the impact of the *Ity3.1* sub-locus on cell death and cytoskeletal reorganization, hematopoiesis as well as propose the candidacy of *Selp* (selectin P) as one of the candidate genes underlying *Ity3.2* based on expression analysis, coding sequence polymorphism, and functional and allelic complementation studies.

## Materials and Methods

### Ethics statement

All animals were maintained at the Animal Care Facility of McGill University according to the guidelines of the Canadian Council on Animal Care (CCAC). The animal protocol for this study was approved by the McGill University Animal Care Committee.

### Animals

Classical inbred strain C57BL/6J and wild-derived MOLF/Ei mice were used to generate congenic, B6.MOLF-*Ity* and B6.MOLF-*Ity/Ity3* and sub-congenic mice as described previously (12, 16). The susceptible *Ity3* and resistant *Ity* mice, as well as the intermediate B6.MOLF-*Ity/Ity3.RecN* and B6.MOLF-*Ity/Ity3.RecG* mice were used for the microarray expression analysis, while the B6.MOLF-*Ity* and B6.MOLF-*Ity/Ity3*, crossed with B6.129S7-*Selp*^tm1Bay^/J ordered from the Jackson Lab (Bar Harbor, ME, USA), were used for the complementation assay.

### *In vivo Salmonella* infection

Mice aged 7–12 weeks were infected with *Salmonella typhimurium* strain Keller as described previously (12, 16). Briefly, mice were inoculated with 0.2 ml of physiological saline containing 10^3^ colony-forming units of bacteria through the caudal vein. The infectious dose was verified by serial dilutions on trypticase soy agar. Mice were either monitored for survival or euthanized at day 3 or day 5 post-infection for organ collection. The animals were monitored two to three times daily and mice showing body condition scoring <2.0 were used for clinical endpoint (17). Survival analysis was conducted using a Kaplan–Meier survival test.

### Microarray expression analysis

RNA was extracted from the spleens of mice, which were collected before infection and at day 3 post-infection. The RNA extraction was carried out using TRIzol reagent (Invitrogen Canada, Inc., Burlington, ON, Canada). Three age-matched male mice were used per group. The concentration of RNA was determined using a NanoDrop spectrophotometer (Thermo-Fisher Scientific, Waltham, MA, USA). All hybridization and scanning of mice microarrays were carried out at the McGill University and Genome Quebec Innovation Centre, using the Illumina BeadArray technology (Illumnia Inc., San Diego, CA, USA). The expression data were analyzed using FlexArray and normalized using a Lumi algorithm (Illumnia). Following the normalization, two approaches were used to generate a list of genes differentially expressed across the various strains. First, a Cyber *t*-test (Baladi and Long) (18) was used to generate a list of genes differentially expressed during infection, by comparing the fold change in expression of each gene between infected and uninfected samples for each strain. These gene lists were further refined using the Benjamini Hochberg false discovery rate algorithm. Genes with an FDR *p*-value <0.1 and a fold change of >2 were used to generate a final list of genes, which represented genes differentially regulated in each strain upon infection.

A second approach was used to generate a list of genes that were differentially expressed at each time point, as compared to the control *Ity* strain. This was done by comparing the expression of genes in each strain to the control *Ity* strain at both day 0 and day 3 post-infection. The gene lists were further refined using the Benjamini Hochberg false discovery rate algorithm. Genes with an FDR *p*-value of <0.05 was used as a cut-off to characterize genes as significantly differentially regulated as compared to *Ity*. The gene lists generated using the two approaches were studied using a suite of online tools including DAVID (19), GeneGo (MetaCore, Thomson Reuters) and Gene Mania (20, 21).

### Sequencing of *Selp*

Sequencing was performed on PCR-amplified cDNA from *Ity* and *Ity3* congenics obtained by reverse transcription of Trizol spleen-extracted RNA to determine genetic variation between C57BL/6J and MOLF/Ei alleles of the *Selp* candidate gene. Sanger sequencing was completed at the McGill University and Génome Québec Innovation Centre.

### Allelic complementation assay

In order to study the effect of a MOLF/Ei *Selp* allele on susceptibility to infection, we carried out a complementation cross. *Selp* knock-out mice, B6.129S2-*Selp*^tm1Hyn^/J (*Selp*^−/−^) were ordered from the Jackson Laboratories (Bar Harbor, ME, USA). These mice were on C57BL/6J background with a mutant *Slc11a1* allele. In order to correct for this, we crossed the *Selp*^−/−^ mice to *Ity* as well as to the *Ity3* mice. Mice were genotyped for the *Selp*^−/−^ allele, and mice carrying the MOLF/Ei *Slc11a1* allele along with the *Selp*^−/−^ allele were inter-crossed to generate homozygous *Selp*^−/−^ mice with a MOLF/Ei allele at the *Slc11a1* gene. Furthermore, these mice were crossed with *Ity* or *Ity3* mice to generate mice, which are homozygous wild-type at *Slc11a1*, but carry a *Selp* knock-out allele complemented by either a C57BL/6J or MOLF/Ei allele (Figure S1 in Supplementary Material).

### Bacterial load enumeration

For bacterial burden quantification in the spleen and the liver, mice were euthanized using CO_2_ and at the required day post-infection; both organs were removed aseptically, weighed and homogenized using a Polytron (Kinematica, Bohemia, NY, USA). The resulting homogenate was diluted in 0.9% saline and plated on tryptic soy agar to determine organ bacteria burden.

### Statistical analysis

Statistical analysis was performed using Graph Pad Prism 6 (GraphPad Software, San Diego, CA, USA). One-way ANOVA with Dunnet’s multiple correction test was used to analyze the bacterial burden in the spleen. A corrected *p*-value <0.05 was used to establish significant differences.

## Results

### *Ity3* influences the expression of specific genes and pathways during infection

**Figure 1:**
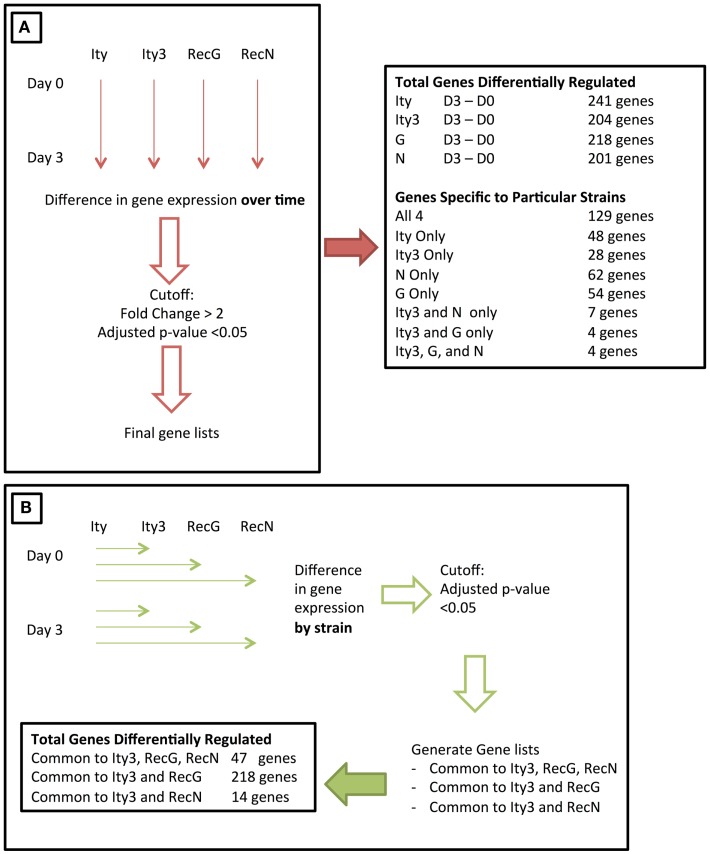
**Figure illustrating the two approaches used to study the changes in gene expression during infection of congenic and sub-congenic mice**. **(A)** A representation of an initial approach used for studying the change in gene expression over time. The gene expression at day 3 post-infection was compared to day 0, and the final number of genes differentially regulated is stated on the right. **(B)** The second approach used, where gene expression in each strain was compared to the control *Ity* strain, at both day 0 and day 3. The final number of genes within each category is shown at the bottom of the panel.

The *Ity3* locus is a complex QTL containing at least two sub-loci. We have previously studied the phenotypic impact of these two sub-loci (16), and in order to further characterize the genetic networks that are affected by the MOLF/Ei allele at the *Ity3* locus, we have used a microarray expression approach. Using this approach, we should be able to characterize and identify candidate genes within each sub-loci and identify pathways, which affect the host immune response to *Salmonella* infection. We studied the splenic global expression profile of infected and uninfected congenic resistant B6.MOLF-*Ity* (*Ity*), susceptible B6.MOLF-*Ity/Ity3* (*Ity3*), and *Ity3.RecG* and *Ity3.RecN* strains. These two sub-congenic strains were selected because they carry either the MOLF/Ei alleles at *Ity3.1* (*Ity3.RecG*) or at *Ity3.2* sub-locus (*Ity3.RecN*), which result in an intermediate survival phenotype after infection with *Salmonella typhimurium* (16). *Ity, Ity3, Ity3.RecN*, and *Ity3.RecG* mice show significant differences in spleen and liver bacterial burden by day 5 post-infection (16). To evaluate primary defects in gene expression rather than differences due to high bacterial load, we selected day 3 as the time point to be studied because there was no significant difference in the spleen bacterial burden among the four strains of mice (16). Therefore, any changes in gene expression will serve as an indicator for genes and pathways that are important in regulating the susceptibility to *Salmonella* infection and not a consequence of a difference in bacterial load within strains. Our aim was to identify transcriptional signatures common to both *Ity*3 and *Ity3.RecG* to define pathways controlled by the *Ity3.1* locus and those similarly regulated by *Ity3* and *Ity3*.*RecN* to identify transcriptional networks controlled by the *Ity3.2* locus. We used two approaches to study the gene expression profiles (Figure 1).

As an initial approach, we studied the changes in splenic gene expression in each strain over time (Day 3–Day 0). Overall, 241 (*Ity*), 204 (*Ity3*), 218 (*Ity3.RecG*), and 201 (*Ity3.RecN*) genes were differentially expressed during infection as defined by a cut-off of a fold change >2 and a *p*-value <0.1 (Tables S1A–D in Supplementary Material; Figure 1). We identified 129 genes that were commonly and significantly regulated during infection in all four strains including a number of pro-inflammatory genes (Table S1E in Supplementary Material). The majority of these genes were chemokines, cytokines, and other immune related genes with a significant number of genes regulated by Type I and Type II IFN including several members of the *Gbp* family, *Stat1, Usp18* as well as others that are known to be involved in *Salmonella* infection (7, 15). These genes (Table S1E in Supplementary Material) define a transcriptional signature that is common during *Salmonella* infection and has been previously detected in different strains of mice during infection (15, 22). Additional strain-specific genes regulated during infection were detected only in *Ity* (40 genes), *Ity3* (20 genes), *Ity3.RecN* (62 genes), and *Ity3.RecG* (54 genes) (Tables S1F–I in Supplementary Material). A large number of differentially expressed genes specific to *Ity* mice (Table S1F in Supplementary Material) were up-regulated in granulocytes and/or macrophages including *S100a8* and *S100a9* that are of particular interest as they are involved with expression of inflammatory mediators, phagocytosis, oxidative burst as well as migration of neutrophils and monocytes to the site of infection (23). The gene *Clec7a* (*dectin 1*) was differentially regulated only in *Ity*. Recent work has linked dectin 1/Syk kinase signaling with autophagy-dependent maturation of phagosomes (24).

In *Ity3* mice, most genes that were differentially regulated were either expressed in macrophages or in megaerythrocyte precursors suggesting active extramedullary erythropoiesis in this strain (Table S1G in Supplementary Material). Interestingly, a large proportion (~40%) of genes specific to the strain *Ity3.RecG* are known to be down-regulated in B and T cells, as analyzed by BioGPS (25) (Table S1G in Supplementary Material). These data show the impact of *Ity3* on the cellular composition of the spleen and/or changes in gene expression in specific cellular populations during infection. Very few genes were similarly regulated between *Ity3* and the sub-congenic strains *Ity3.RecG* (4 genes), *Ity3.RecN* (7 genes), and *Ity3.RecN* and *Ity3.RecG* (4 genes) (Tables S1J–L in Supplementary Material).

**Figure 2:**
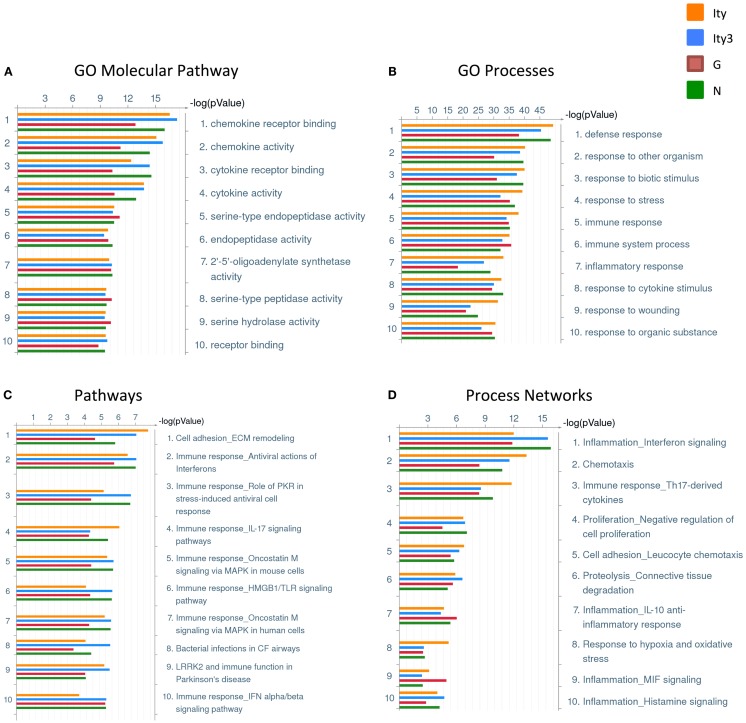
**Pathway and process analyses of genes differentially regulated in the spleen of *Ity, Ity3, Ity3.RecG*, and *Ity3.RecN* mice during infection**. Gene ontology classifications generated by clustering the genes that are differentially regulated in each strain upon infection. **(A)** Gene ontology (GO) molecular pathways and **(B)** Gene ontology (GO) processes **(C)** pathways **(D)** Process networks that are enriched in the four mouse strains. The sub-congenic strain *Ity3.RecG* shows a lower −log(*p*-value) for chemokine and cytokine receptor activity within the GO molecular pathways as well as lower −log(*p*-values) for response to bacteria in the GO processes. This is consistent with previous data suggesting that the *Ity3.RecG* mice have a diminished inflammatory response following infection compared to parental strains (16).

We then used GeneGO (Thomson Reuters, NY, USA) to classify the genes differentially regulated in each strain (Tables S1A–D in Supplementary Material) into gene ontology (GO) molecular pathways, GO processes, pathways, and process networks, in order to identify the pathways differentially regulated in each strain during infection (Figure 2). The strain *Ity3.RecG* appeared to have fewer genes involved in various pathways and processes related to chemokine and cytokine activity and immune response as demonstrated by the lower –log_2_ (*p*-values). These results are consistent with previous observations of reduced inflammatory responses following *in vivo Salmonella* infection in *Ity3.RecG* mice (16).

### *Ity3.1* locus influences the expression of genes involved in cell-cycle regulation and hematopoiesis

**Figure 3:**
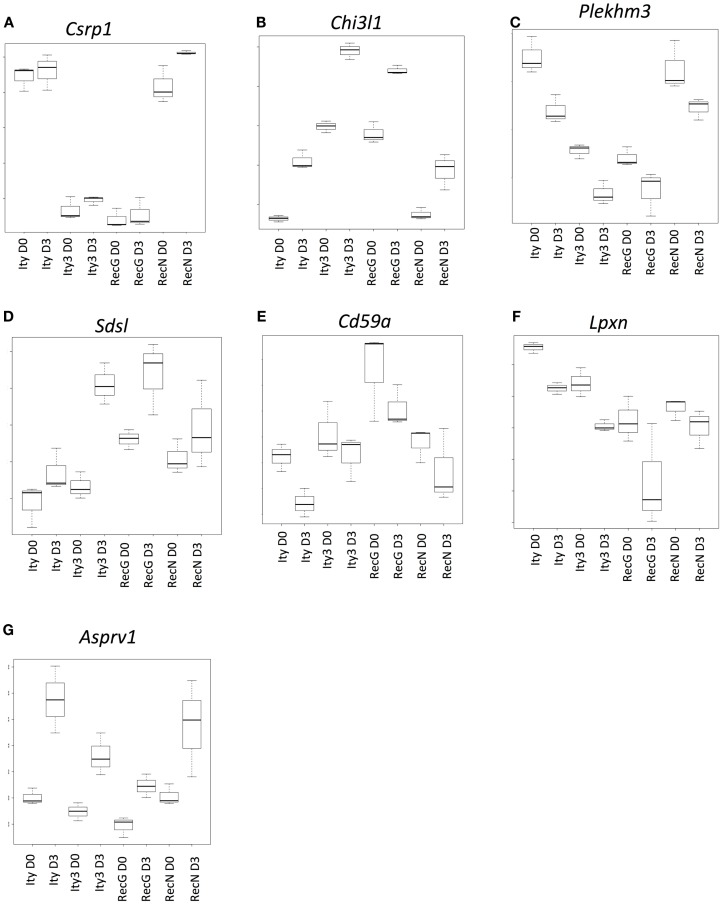
**Genes that are under the control of the *Ity3.1* locus**. Sample box plots of the gene lists provided in Table S2 in Supplementary Material are shown. **(A)** Represents genes, which do not show any changes in expression during infection and show a similar expression pattern in *Ity3* and *Ity3.RecG*. **(B,C)** Represents a sample box plot of gene expression, which show a similar regulation pattern in *Ity3* and *Ity3.RecG*. **(D–G)** Also illustrate genes within Tables S2D,G in Supplementary Material, which show similar expression patterns in *Ity3* and *Ity3.RecG*, but different from *Ity* and *Ity3.RecN*. These genes are likely under the control of the *Ity3.1* locus. *Csrp1* (cysteine and glycine-rich protein 1), *Chi3l1* (chitinase-like 1), (*Plekhm3*) Pleckstrin homology domain containing, family M, member 3), *Sdsl* (serine dehydratase-like), *Cd59a* (CD59a antigen), *Lpxn* (leupaxin), *Asprv1* (aspartic peptidase, retroviral-like 1).

We have previously reported that *Ncf2* is a strong candidate for the *Ity3.1* locus (13). To further characterize the downstream impact of *Ity3.1* and *Ncf2* on gene expression, we analyzed the data by evaluating variation in gene expression between *Ity3, Ity3.RecG*, or *Ity3.RecN*, and the control strain *Ity* at day 0 and day 3 post-infection (Figure 1B). Interestingly, 12 genes located within the introgressed *Ity3* region had lower expression levels in *Ity3* and *Ity3.RecG*, compared to both *Ity* and *Ity3.RecN*. Figure 3A shows the box plot expression pattern of one of these genes and the list is provided in Table S2A in Supplementary Material. None of these genes were differentially regulated during infection in any of the sub-congenic strains. For some of these genes, the low levels of expression detected in mouse strains *Ity3* and *Ity3.RecG* appear to be a consequence of poor hybridization of MOLF/Ei cDNA to microarray probes as a result of high genomic variability between the MOLF/Ei and C57BL/6J strains (Figure S2 in Supplementary Material). The wild-derived inbred MOLF/Ei had been separated from the classical inbred trains by over 1 million year of evolution, and as a result they have accumulated significant sequence variability, to the order of 1 SNP every 100 bp (26, 27).

A second set of genes showed similar patterns of up-regulation (Figure 3B; Table S2B in Supplementary Material) or down-regulation (Figure 3C; Table S2C in Supplementary Material) during infection in all strains, although the constitutive and induced expressions were similar in *Ity3* and *Ity3.RecG* but significantly different from *Ity* and *Ity3.RecN*. This expression pattern highlights the impact of the MOLF/Ei allele at the *Ity3* locus, as all the genes, which an expression pattern similar to Figures 3B,C carry a MOLF/Ei allele at the *Ity3.1* locus (16).

Another set of genes showed higher expression during infection only in strain *Ity3* and *Ity3.RecG* (Figures 3D,E; Tables S2D,E in Supplementary Material) as compared to *Ity*. In contrast to this grouping, the cluster of genes in Figure 3F exhibit lower expression levels in *Ity3* and *Ity3.RecG*. Collectively, these groups of genes in Figures 3D–G (Tables S2D–G in Supplementary Material) exhibit a pattern of expression that is similar between *Ity3* and *Ity3.RecG*, and the expression differences could not be attributed solely to the MOLF/Ei allele at chromosome 1, therefore, we can conclude that the expression differences are likely under the influence of the *Ity3.1* locus.

Functional annotation of genes, which have an expression pattern similar between *Ity3* and *Ity3.RecG* (Tables S2G–D in Supplementary Material) showed that a large percentage of the genes in the list plays a role in cell cycle, DNA binding, cytoskeletal reorganization, and hemopoietic and lymphoid organ development (Table S3 in Supplementary Material) (19, 28). Another major category of genes such as *Ank1* and *Uros* are involved in heme metabolic process. These data are consistent with the observation that ROS control cell-cycle progression by influencing the presence and activity of cyclins and cyclin dependent kinases (29) and with a role for ROS in maturation and lifespan of erythroid cells (30, 31).

### *Ity3.1* affects a group of genes that are co-expressed with *Ncf2*

**Figure 4:**
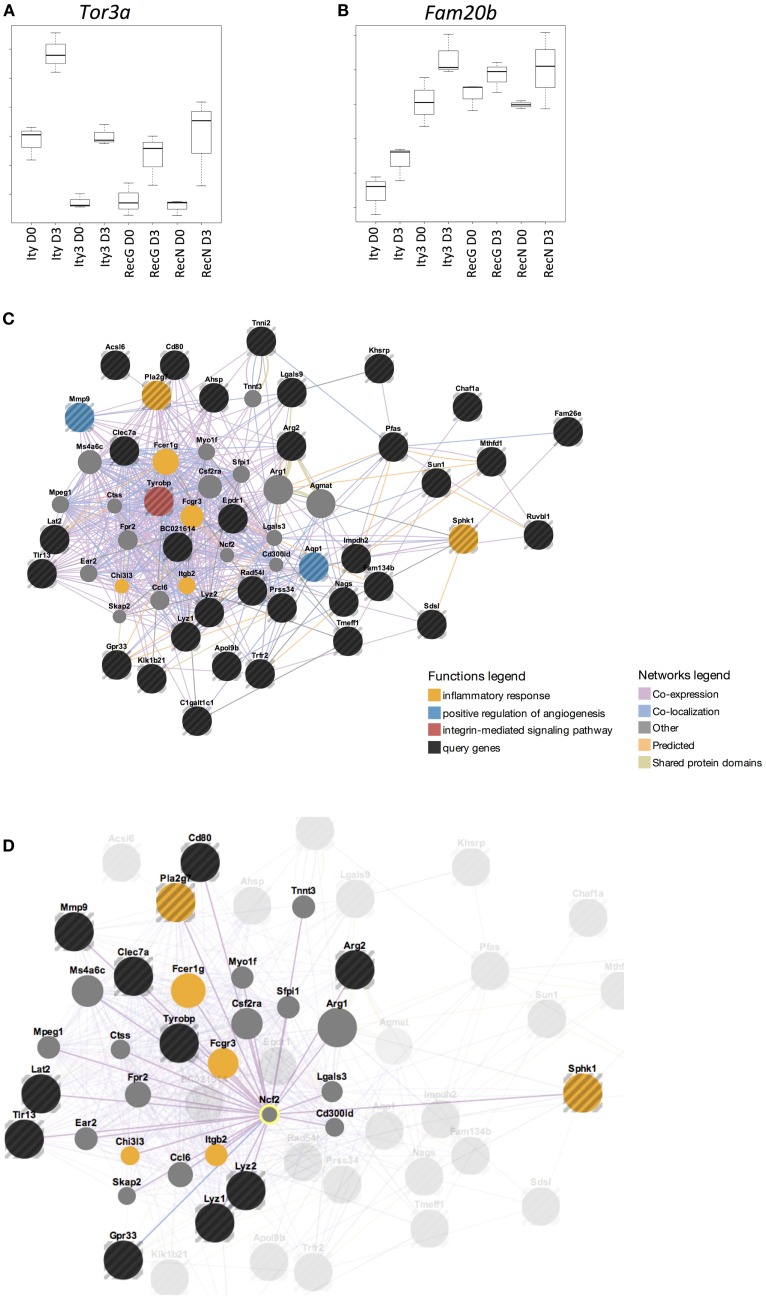
**Genes that are differentially expressed in all susceptible and intermediate strains are under the influence of *Ity3.1* sub-locus**. Box plots of the expression pattern of two genes **(A)** *Tor3a* (torsin family 3, member A) and **(B)** *Fam20b* (family with sequence similarity 20, member B) are shown as examples to illustrate the expression pattern seen in the gene list provided in Table S2I in Supplementary Material. This list of genes show a similar expression in *Ity3, Ity3.RecG*, and *Ity3.RecN* and highlights the complex nature of the *Ity3* locus as there are multiple genes in which expression is influenced by the combination of the two sub-loci. The genes that show a similar expression pattern in *Ity3, Ity3.RecG*, and *Ity3.RecN* were studied using GeneMania and the results are shown **(C,D)**, with the query genes being highlighted in black. Genes, which are known to be co-expressed, co-localized, have shared domains or predicted interactions with the list of genes in Table S2I in Supplementary Material are shown. The functional categories, which are enriched within this gene list, are inflammatory response, angiogenesis, and integrin mediated signaling pathways shown in yellow, blue, and red, respectively. Genes that were not differentially expressed but important in these pathways are shown in gray. **(D)** Co-expression of query genes, as well as other genes within these pathways, with *Ncf2* is shown.

In order to define the gene expression profile of the susceptible strains, we identified genes that had a similar pattern of expression in the susceptible strain, *Ity3* as well as the two sub-congenic strains *Ity3.RecN* and *Ity3.RecG*. Figures 4A,B highlights two genes, *Tor3a* and *Fam20b* as examples of the expression pattern of the list of genes provided in Table S2H in Supplementary Material, which have a similar expression pattern in *Ity3, Ity3.RecN*, and *Ity3.RecG*. Only 7 of the 47 genes were within the *Ity3* interval, and almost all of them were within the genomic region common to *Ity3.RecN* and *Ity3.RecG*. This gene list was classified within functional categories (Figure 4C) such as inflammatory response and regulation of angiogenesis. A large proportion of the genes were either co-expressed, co-localized, or have shared domains or predicted interactions with *Ncf2* (Figure 4D). We have previously shown that the MOLF/Ei allele at the *Ity3.1* locus contributed the strongest effect on susceptibility to *Salmonella* infection and was responsible for high bacterial burden and low ROS and cytokine production (16). The fact that a number of genes differentially regulated in *Ity3, Ity3.RecN*, and *Ity3*.*RecG* strains, were co-expressed with *Ncf2*, supports the important contribution of the *Ity3.1* locus on the pathogenesis of infection in MOLF/Ei and its interaction with the other sub-locus *Ity3.2* to enhance the impact of *Ncf2* on ROS production.

### *Selp* is a candidate gene for *Ity3.2*

**Figure 5:**
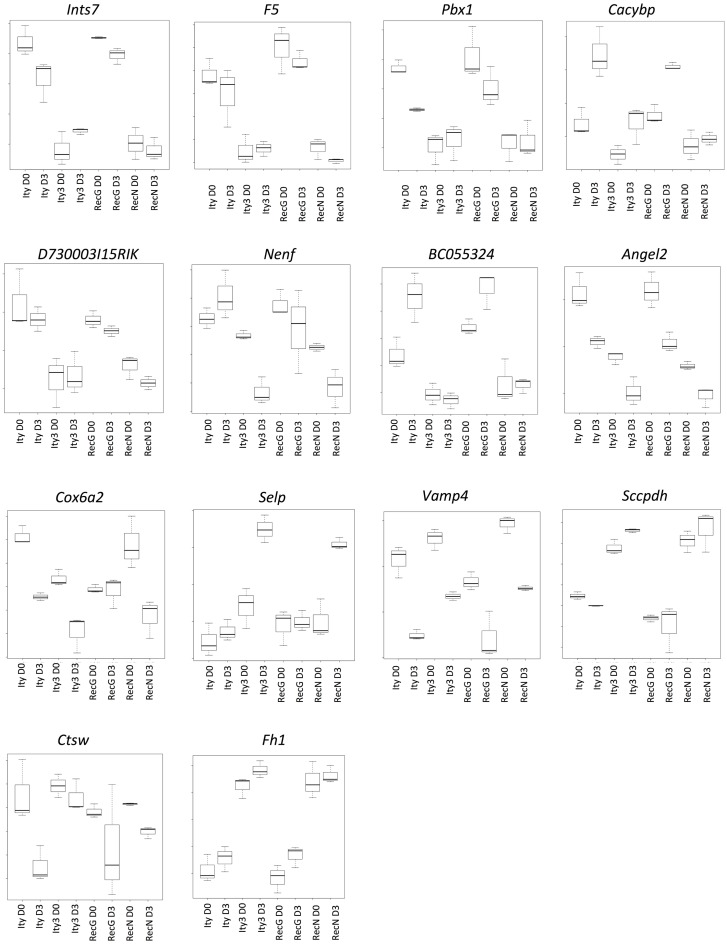
**Genes under the influence of *Ity3.2***. List of genes showing a similar expression pattern in *Ity3* and *Ity3.RecN*, and different from *Ity* and *Ity3.RecG. Ints7* (integrator complex subunit 7), *F5* (coagulation factor V), *Pbx1* (pre B cell leukemia homeobox 1), *Cacybp* (calcyclin binding protein), *Nenf* (neuron derived neurotrophic factor), *Angel2* [angel homolog 2 (*Drosophila*)], *Cox6a2* (cytochrome c oxidase subunit VIa polypeptide 2), *Selp* (selectin, platelet), *Vamp4* (vesicle-associated membrane protein 4), *Sccpdh* [saccharopine dehydrogenase (putative)], *Ctsw* (cathepsin W), *Fh1* (fumarate hydratase 1).

We next studied genes showing a similar regulation pattern in *Ity3* and *Ity3.RecN* to understand the pathways differentially regulated in *Ity3.2* and identify potential candidate genes for the *Ity3.2* locus. There were only 14 genes that showed a similar expression pattern in *Ity3* and *RecN* (Figure 5). Of these 14 genes, 12 are located on chromosome 1 and 6 genes (*F5, Pbx1, Cacybp, Bc055342, Selp*, and *Vamp4*) lie within the genomic region harboring *Ity3.2* (Table 1). Sequence variations have been reported between the MOLF/Ei and C57BL/6J in coagulation factor *F5*, the cDNA *BC055324* and selectin P (*Selp*). The coagulation factor V is synthesized by the liver and is involved in the acceleration of prothrombin to thrombin conversion (32). Coagulation Factor V deficiency leads to a bleeding disorder associated with mild to severe hemorrhagic symptoms (33). The cDNA *BC055324* is poorly characterized and its function is not known. The *Selp* gene encodes for an adhesion molecule that mediates the recruitment of immune cells to the site of inflammation and is critical for the host immune response to infection making this gene an attractive candidate gene for *Ity3.2*.

**Table 1:** **List of known SNPs within differentially expressed genes in *Ity3* and *Ity3.RecN***.

Target	Variation	Chromosome	Location (bp)
**DOWN-REGULATED**			
*Ints7*	No exonic variation	1	191575734
*F5*	rs6271495	1	164151838
*Pbx1*	No exonic variation	1	168153527
*Cacybp*	No exonic variation	1	160202367
*D730003i15rik*	No exonic variation	1	191224474
*Nenf*	No exonic variation	1	191306789
*Bc055324*	rs30651611	1	163945993
*Angel2*	No exonic variation	1	190925112
*Cox6a2*	No exonic variation	7	128205436
**UP-REGULATED**			
*Selp*	rs30667849	1	164115264
*Fh1*	rs13465421	1	175600374
*Vamp4*	No exonic variation	1	162570515
*Sccpdh*	No exonic variation	1	179668210
*Ctsw*	No exonic variation	9	5465240

*Table based on data from the Mouse Genome Informatics (MGI) website*.

**Figure 6:**
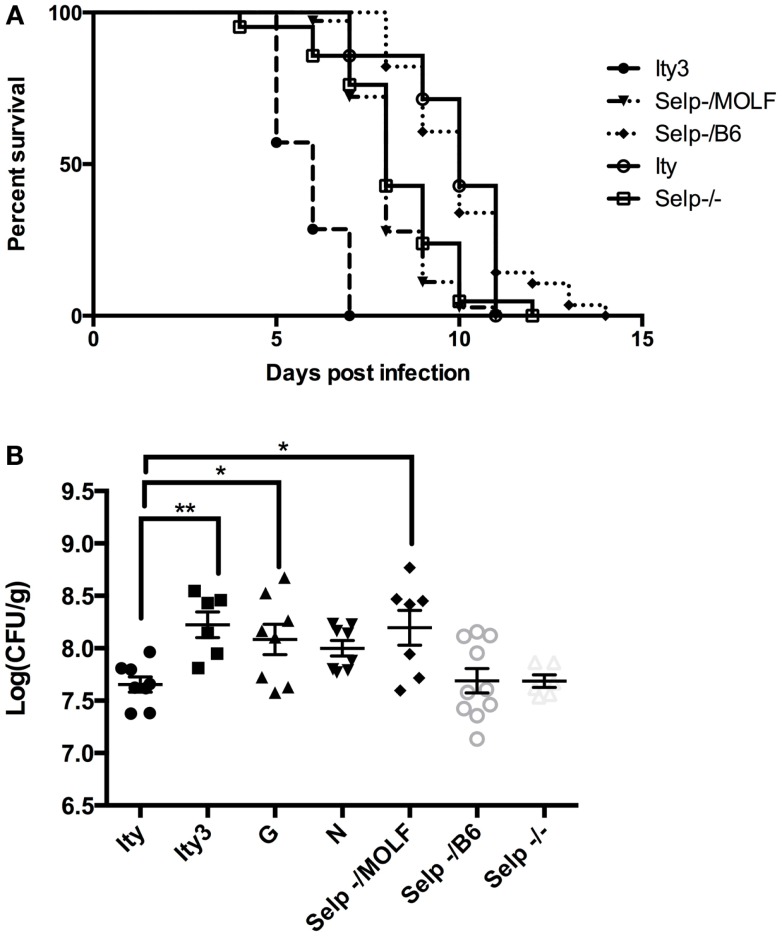
**Allelic complementation between *Ity3* congenic mouse and *Selp* deficient mice**. In order to assess the impact of the MOLF/Ei *Selp* locus, *Selp*^−/−^ mice were crossed with *Ity* and *Ity3* mice to generate mice carrying the knock-out allele complemented by the MOLF/Ei allele at *Selp* or C57BL/6J allele at the *Ity3* locus. **(A)** Survival curves of the congenic *Ity* (*n* = 7), *Ity3* (*n* = 7), knock-out *Selp*^−/−^ (*n* = 21), and *Selp*^−/MOLF^ (*n* = 36), *Selp*^−/B6^ (*n* = 56) mice after infection with *Salmonella typhimurium*. The *Selp*^−/−^ and *Selp*^−/MOLF^ show a similar curve after infection with *Salmonella typhimurium*, both of which are more susceptible than the control *Ity* congenic mice and *Selp*^−/B6^, but more resistant than the mice carrying the entire *Ity3* locus (*Ity3*). **(B)** Bacterial burden in the spleen of congenic *Ity* (*n* = 8) and *Ity3* (*n* = 6), sub-congenic *Ity3.RecG* (*n* = 8), and *Ity3.RecN* (*n* = 8) and *Selp*^−/−^ (*n* = 6) and compound heterozygous (*n* = 7 for *Selp*^−^*^/MOLF^* and *n* = 10 for *Selp*^−/^*^B6^* mice after infection).

We further evaluated the candidacy of *Selp* as the gene underlying *Ity3.2* using sequence analysis and complementation assay *in vivo*. *Selp* encodes for a protein of 768 amino acids with a C-type lectin domain, an EGF domain and 8 complement control protein (CCP) modules [or as short consensus repeats (SCRs) functional domains]. We re-sequenced the coding region of *Selp* in C57BL/6J and MOLF/Ei mice and identified eight SNPs (Table 2), all of which are within the less homologous CCP domains, involved in protein recognition processes (34). The amino acid proline at position 205 is well conserved across 12 mammalian species and P205S is only observed in the DBA sub-strains, which share MOLF/Ei ancestry for this region of the genome (mouse phylogeny viewer) (35). In order to validate that the sequence variation in the MOLF/Ei *Selp* gene has an impact on susceptibility to infection with *Salmonella typhimurium* and to evaluate if *Selp* is indeed the gene underlying *Ity3.2*, we used an allelic complementation assay (see breeding scheme in Figure S1 in Supplementary Material). *Ity* (*Selp^B6/B6^*) and *Ity3* (*Selp^MOLF/MOLF^*) mice were crossed to *Selp*^−/−^ knock-out mice and susceptibility to infection was assessed by survival analysis in F1 progeny with *Selp^MOLF/^*^−^ and *Selp^B6/^*^−^ genotypes. *Selp^MOLF/^*^−^ mice were significantly more susceptible to infection than *Selp^B6/^*^−^ mice and *Ity* controls (Figure 6A). We observed a lack of complementation in *Selp^MOLF/^*^−^ mice with a mean survival time equivalent to *Selp*^−/−^ animals (MST of 8.1 ± 0.18 and 8.3 ± 0.38, respectively), adding further support for the candidacy of *Selp* as the gene underlying the *Ity3.2* locus (Figure 6A). Although the *Selp*^−/−^ mice showed a similar susceptibility compared to the *Selp^MOLF^*^/−^ mice in terms of survival, their tissue bacterial burden was significantly lower when compared to *Selp^MOLF^*^/−^, *Ity3*, and *RecG* mice (Figure 6B) suggesting that the *Ity3*.2 locus does not contribute significantly to the bacterial burden and that the high bacterial burden observed in *Selp^MOLF^*^/−^ mice is rather the effect of the *Ity3.1* locus.

**Table 2:** **Exonic variation in the MOLF/Ei allele of the *Selp* gene**.

	Base pair change	Amino acid change	Domain
*Selp*	730G > T	V202F	Sushi/CCP/SCR
	739A > G	N203D	Sushi/CCP/SCR
	745C > T	P205S	Sushi/CCP/SCR
	620C > T	H207Y	Sushi/CCP/SCR
	841G > A	G239S	Sushi/CCP/SCR
	1135G > A	V337I	Sushi/CCP/SCR
	1775A > C	N550T	Sushi/CCP/SCR
	1831A > G	I569V	Sushi/CCP/SCR

## Discussion

The current study was specifically designed to understand the pathways that are influenced by the *Ity3* locus using sub-congenic strains that exhibit different degrees of susceptibility to *Salmonella* infection. The global gene expression profile in the spleen was studied early during infection prior to a significant bacterial difference in the target tissue. This approach allowed us to identify networks, which are of importance in the early phases of innate immunity yet not influenced by the extent of bacterial burden in the spleen. We reported a group of genes, the majority of which are regulated by type I and type II IFN. These genes, such as the *Gbp, Oas, Ifitm* family members, are differentially regulated in all strains of mice during infection, and define a core transcriptional signature common to several strains of mice infected with *Salmonella* (15).

Additionally, we characterized a number of genes not located within the *Ity3* region that were differentially expressed in the susceptible *Ity3, Ity3*.*RecG*, and *Ity3*.*RecN* strains as compared to the resistant *Ity* strain. We showed that these genes are also co-expressed with *Ncf2*, further supporting the hypothesis that there is an influence of the *Ity3.1* sub-locus on other segments of the genome. These results highlight the importance of the region of distal chromosome 1 carrying *Ity3*, a region enriched in QTLs. Over 80 QTLs are listed at the mouse genome database (36) and a number of cis and trans eQTLs have been characterized (37). Among them, several QTLs are involved with complex inflammatory reaction, such as graft vs. host disease (38) lupus (39), modifier of LPS-response (40), and susceptibility to tuberculosis (41).

We have reported previously that *Ity3* influences ROS production during infection (13) and this effect was mapped recently to a small sub-region named *Ity3.1*, which harbors the gene *Ncf2*, a subunit of the NADPH complex. (16). ROS produced by NADPH has been shown to affect a number of pathways, which are important in innate immunity including bacterial killing within the phagolysosome where ROS interact with other ions such as chloride to form toxic agents (HOCL) or can convert into hydroxyl radicals that are toxic for bacteria (42). ROS production has also been shown to influence immune cell recruitment, activation, and survival (43), and lead to translational activation of NF-κB (44). NADPH oxidase derived ROS is also a key regulator of autophagy and autophagy regulation during pathogen invasion (45). In addition, NADPH oxidase activation contributes to the recognition and removal of apoptotic neutrophils (efferocytosis) by macrophages (46–48). Therefore, an imbalance in NADPH produced ROS can lead to increased inflammation, which can be deleterious to the host.

Recent studies have shown that TNF, as well as other cytokines through NF-κB signaling induces transient increase in ROS level in endothelial cells, which results in cell surface expression of *Icam* and *Selp* (49–52). This effect has further been studied *in vivo*, where mice lacking the p47^phox^ (*Ncf1*) subunit of the NADPH complex have reduced expression of VCAM-1, ICAM-1, SELP, and SELE in the vascular cell walls (53, 54). In the current study, we identified *Selp* as a candidate gene for the locus *Ity3.2* and used an allelic complementation assay to provide genetic evidences that *Selp* is indeed a strong candidate for the *Ity3.2* locus. In MOLF/Ei mice susceptibility to infection as explained by the *Ity3* locus could be attributed to the individual effect of *Ity3.1* and *Ity3.2* sub-loci but also to the cooperation between these two sub-loci as explained by the potential impact of low activity of NADPH oxidase (16) on *Selp* function by reducing its expression (current paper).

We also illustrated that mice carrying a MOLF/Ei allele at the *Ity3.1* locus have higher expression of a number of genes playing a role in cell cycle, DNA binding, and cytoskeletal pathways. There is a growing body of evidence discussing the link between ROS, cell-cycle progress and arrest. As discussed by Martindale (55), ROS can have multiple effects on the cell cycle, depending on the amount and type of ROS. They suggest that low doses of ROS may cause proliferation while high doses of ROS can lead to apoptosis and cell death. In our model, it is possible that reduced levels of ROS in susceptible animals could lead to cell growth arrest, hence providing a more favorable niche for bacteria to replicate.

Another pathway influenced by ROS production, is up-regulated by *Ity3.1* and regroups genes involved in heme biosynthesis. Increased expression of genes within the heme biosynthesis pathway could result in increased free heme within the cells, which can act as a potent cytotoxic pro-oxidant (56). Free heme has also recently been shown to trigger necroptosis in macrophages (57). Therefore, it is possible that the increased expression of the heme biosynthesis pathway observed in susceptible mice is a mechanism that compensates for the low ROS levels.

In conclusion, our study highlights the complex nature of multi-loci interaction in the wild-derived MOLF/Ei response to *Salmonella* infection. We highlighted the role of low ROS and cytokine production in reduced survival of mice carrying the *Ity3.1* locus, and the importance of the *Ity3.2* locus, which synergistically led to increased susceptibly of the *Ity3* mice. We have also shown that several pathways identified in strains *Ity3, Ity3.RecN*, and *Ity3.RecG*, are influenced by *Ncf2*. Furthermore, the *Ity3.1* sub-locus has additional effects, which have not previously been characterized, in expression of genes involved in cell-cycle arrest and hematopoiesis. Lastly, we propose a hypothesis that the combined effects of low ROS production by the MOLF/Ei *Ity3.1* locus together with the impact of *Selp* MOLF/Ei allele at *Ity3.2* influences the host survival after infection with *Salmonella typhimurium*.

## Conflict of Interest Statement

The authors declare that the research was conducted in the absence of any commercial or financial relationships that could be construed as a potential conflict of interest.

## Supplementary Material

The Supplementary Material for this article can be found online at http://www.frontiersin.org/Journal/10.3389/fimmu.2014.00375/abstract

## References

[1] Mastroeni P Grant A Dynamics of spread of *Salmonella enterica* in the systemic compartment. Microbes Infect (2013) 15:849–57 10.1016/j.micinf.2013.10.003 24183878

[2] Mastroeni P Grant A Restif O Maskell D A dynamic view of the spread and intracellular distribution of *Salmonella enterica*. Nat Rev Microbiol (2009) 7:73–80 10.1038/nrmicro2034 19079353

[3] Buckle GC Walker CL Black RE Typhoid fever and paratyphoid fever: systematic review to estimate global morbidity and mortality for 2010. J Glob Health (2012) 2:10401 10.7189/jogh.02.010401 23198130 PMC3484760

[4] de Jong HK Parry CM van der Poll T Wiersinga WJ Host-pathogen interaction in invasive salmonellosis. PLoS Pathog (2012) 8:e1002933 10.1371/journal.ppat.1002933 23055923 PMC3464234

[5] Mastroeni P Sheppard M *Salmonella* infections in the mouse model: host resistance factors and in vivo dynamics of bacterial spread and distribution in the tissues. Microbes Infect (2004) 6:398–405 10.1016/j.micinf.2003.12.009 15101397

[6] Sebastiani G Olien L Gauthier S Skamene E Morgan K Gros P Mapping of genetic modulators of natural resistance to infection with *Salmonella typhimurium* in wild-derived mice. Genomics (1998) 47:180–6 10.1006/geno.1997.5116 9479490

[7] Richer E Yuki KE Dauphinee SM Larivière L Paquet M Malo D Impact of Usp18 and IFN signaling in *Salmonella*-induced typhlitis. Genes Immun (2011) 12:531–43 10.1038/gene.2011.38 21614019

[8] Roy MF Riendeau N Bédard C Hélie P Min-Oo G Turcotte K Pyruvate kinase deficiency confers susceptibility to *Salmonella typhimurium* infection in mice. J Exp Med (2007) 204:2949–61 10.1084/jem.20062606 17998386 PMC2118530

[9] Vidal S Gros P Skamene E Natural resistance to infection with intracellular parasites: molecular genetics identifies Nramp1 as the Bcg/Ity/Lsh locus. J Leukoc Biol (1995) 58:382–90 7561513 10.1002/jlb.58.4.382

[10] Sebastiani G Blais V Sancho V Vogel SN Stevenson MM Gros P Host immune response to *Salmonella enterica* serovar typhimurium infection in mice derived from wild strains. Infect Immun (2002) 70:1997–2009 10.1128/IAI.70.4.1997-2009.2002 11895964 PMC127833

[11] Sebastiani G Leveque G Larivière L Laroche L Skamene E Gros P Cloning and characterization of the murine toll-like receptor 5 (Tlr5) gene: sequence and mRNA expression studies in *Salmonella*-susceptible MOLF/Ei mice. Genomics (2000) 64:230–40 10.1006/geno.2000.6115 10756091

[12] Sancho-Shimizu V Khan R Mostowy S Larivière L Wilkinson R Riendeau N Molecular genetic analysis of two loci (Ity2 and Ity3) involved in the host response to infection with *Salmonella typhimurium* using congenic mice and expression profiling. Genetics (2007) 177:1125–39 10.1534/genetics.107.075523 17660555 PMC2034618

[13] Sancho-Shimizu V Malo D Sequencing, expression, and functional analyses support the candidacy of Ncf2 in susceptibility to *Salmonella typhimurium* infection in wild-derived mice. J Immunol (2006) 176:6954–61 10.4049/jimmunol.176.11.6954 16709856

[14] Roy MF Malo D Genetic regulation of host responses to *Salmonella* infection in mice. Genes Immun (2002) 3:381–93 10.1038/sj.gene.6363924 12424619

[15] Khan R Sancho-Shimizu V Prendergast C Roy MF Loredo-Osti JC Malo D Refinement of the genetics of the host response to *Salmonella* infection in MOLF/Ei: regulation of type 1 IFN and TRP3 pathways by Ity2. Genes Immun (2012) 13:175–83 10.1038/gene.2011.69 21956657

[16] Khan RT Yuki KE Malo D Fine-mapping and phenotypic analysis of the Ity3 *Salmonella* susceptibility locus identify a complex genetic structure. PLoS One (2014) 9:e88009 10.1371/journal.pone.0088009 24505352 PMC3913713

[17] Ullman-Cullere MH Foltz CJ Body condition scoring: a rapid and accurate method for assessing health status in mice. Lab Anim Sci (1999) 49:319–23 10403450

[18] Long AD Mangalam HJ Chan BY Tolleri L Hatfield GW Baldi P Improved statistical inference from DNA microarray data using analysis of variance and a Bayesian statistical framework. Analysis of global gene expression in *Escherichia coli* K12. J Biol Chem (2001) 276:19937–44 10.1074/jbc.M010192200 11259426

[19] Huang da W Sherman BT Lempicki RA Systematic and integrative analysis of large gene lists using DAVID bioinformatics resources. Nat Protoc (2009) 4:44–57 10.1038/nprot.2008.211 19131956

[20] Zuberi K Franz M Rodriguez H Montojo J Lopes CT Bader GD GeneMANIA prediction server 2013 update. Nucleic Acids Res (2013) 41:W115–22 10.1093/nar/gkt533 23794635 PMC3692113

[21] Warde-Farley D Donaldson SL Comes O Zuberi K Badrawi R Chao P The GeneMANIA prediction server: biological network integration for gene prioritization and predicting gene function. Nucleic Acids Res (2010) 38:W214–20 10.1093/nar/gkq537 20576703 PMC2896186

[22] Caron J Larivière L Nacache M Tam M Stevenson MM McKerly C Influence of Slc11a1 on the outcome of *Salmonella enterica* serovar enteritidis infection in mice is associated with Th polarization. Infect Immun (2006) 74:2787–802 10.1128/IAI.74.5.2787-2802.2006 16622216 PMC1459719

[23] Kerkhoff C Voss A Scholzen TE Averill MM Zänker KS Bornfeldt KE Novel insights into the role of S100A8/A9 in skin biology. Exp Dermatol (2012) 21:822–6 10.1111/j.1600-0625.2012.01571.x 22882537 PMC3498607

[24] Kyrmizi I Gresnigt MS Akoumianaki T Samonis G Sidiropoulos P Boumpas D Corticosteroids block autophagy protein recruitment in *Aspergillus fumigatus* phagosomes via targeting dectin-1/Syk kinase signaling. J Immunol (2013) 191:1287–99 10.4049/jimmunol.1300132 23817424 PMC3883106

[25] Wu C Orozco C Boyer J Leglise M Goodale J Batalov S BioGPS: an extensible and customizable portal for querying and organizing gene annotation resources. Genome Biol (2009) 10:R130 10.1186/gb-2009-10-11-r130 19919682 PMC3091323

[26] Abe K Noguchi H Tagawa K Yuzuriha M Toyoda A Kojima T Contribution of Asian mouse subspecies *Mus musculus* molossinus to genomic constitution of strain C57BL/6J, as defined by BAC-end sequence-SNP analysis. Genome Res (2004) 14:2439–47 10.1101/gr.2899304 15574823 PMC534668

[27] Ideraabdullah FY de laCasa-Esperón E Bell TA Detwiler DA Magnuson T Sapienza C Genetic and haplotype diversity among wild-derived mouse inbred strains. Genome Res (2004) 14:1880–7 10.1101/gr.2519704 15466288 PMC524411

[28] Huang da W Sherman BT Lempicki RA Bioinformatics enrichment tools: paths toward the comprehensive functional analysis of large gene lists. Nucleic Acids Res (2009) 37:1–13 10.1093/nar/gkn923 19033363 PMC2615629

[29] Verbon EH Post JA Boonstra J The influence of reactive oxygen species on cell cycle progression in mammalian cells. Gene (2012) 511:1–6 10.1016/j.gene.2012.08.038 22981713

[30] Marinkovic D Zhang X Yalcin S Luciano JP Brugnara C Huber T Foxo3 is required for the regulation of oxidative stress in erythropoiesis. J Clin Invest (2007) 117:2133–44 10.1172/JCI31807 17671650 PMC1934587

[31] Xu Y Swartz KL Siu KT Bhattacharyya M Minella AC Fbw7-dependent cyclin E regulation ensures terminal maturation of bone marrow erythroid cells by restraining oxidative metabolism. Oncogene (2013) 33(24):3161–71 10.1038/onc.2013.289 23873023 PMC3939062

[32] Esmon CT Owen WG Duiguid DL Jackson CM The action of thrombin on blood clotting factor V: conversion of factor V to a prothrombin-binding protein. Biochim Biophys Acta (1973) 310:289–94 10.1016/0005-2795(73)90034-2 4736336

[33] Asselta R Peyvandi F Factor V deficiency. Semin Thromb Hemost (2009) 35:382–9 10.1055/s-0029-1225760 19598066

[34] Libert F Vassart G Structure-function relationships of the complement components. Immunol Today (1989) 10:407 10.1016/0167-5699(89)90036-4 2619880

[35] Wang JR de Villena FP McMillan L Comparative analysis and visualization of multiple collinear genomes. BMC Bioinformatics (2012) 13(Suppl 3):S13 10.1186/1471-2105-13-S3-S13 22536897 PMC3311102

[36] Blake JA Bult CJ Eppig JT Kadin JA Richardson JE Mouse Genome Database Group The mouse genome database: integration of and access to knowledge about the laboratory mouse. Nucleic Acids Res (2014) 42:D810–7 10.1093/nar/gkt1225 24285300 PMC3964950

[37] Mozhui K Ciobanu DC Schikorski T Wang X Lu L Williams RW Dissection of a QTL hotspot on mouse distal chromosome 1 that modulates neurobehavioral phenotypes and gene expression. PLoS Genet (2008) 4:e1000260 10.1371/journal.pgen.1000260 19008955 PMC2577893

[38] Allen RD Dobkins JA Harper JM Slayback DL Genetics of graft-versus-host disease, I. A locus on chromosome 1 influences development of acute graft-versus-host disease in a major histocompatibility complex mismatched murine model. Immunology (1999) 96:254–61 10.1046/j.1365-2567.1999.00626.x 10233703 PMC2326746

[39] Kono DH Park MS Theofilopoulos AN Genetic complementation in female (BXSB x NZW)F2 mice. J Immunol (2003) 171:6442–7 10.4049/jimmunol.171.12.6442 14662843

[40] Matesic LE De Maio A Reeves RH Mapping lipopolysaccharide response loci in mice using recombinant inbred and congenic strains. Genomics (1999) 62:34–41 10.1006/geno.1999.5986 10585765

[41] Kramnik I Dietrich WF Demant P Bloom BR Genetic control of resistance to experimental infection with virulent *Mycobacterium tuberculosis*. Proc Natl Acad Sci U S A (2000) 97:8560–5 10.1073/pnas.150227197 10890913 PMC26987

[42] Yang HC Cheng ML Ho HY Chiu DT The microbicidal and cytoregulatory roles of NADPH oxidases. Microbes Infect (2011) 13:109–20 10.1016/j.micinf.2010.10.008 20971207

[43] Segal BH Grimm MJ Khan AN Han W Blackwell TS Regulation of innate immunity by NADPH oxidase. Free Radic Biol Med (2012) 53:72–80 10.1016/j.freeradbiomed.2012.04.022 22583699 PMC3377837

[44] Schmidt KN Amstad P Cerutti P Baeuerle PA The roles of hydrogen peroxide and superoxide as messengers in the activation of transcription factor NF-kappa B. Chem Biol (1995) 2:13–22 10.1016/1074-5521(95)90076-4 9383399

[45] Huang J Canadien V Lam GY Steinberg BE Dinauer MC Magalhaes MA Activation of antibacterial autophagy by NADPH oxidases. Proc Natl Acad Sci U S A (2009) 106:6226–31 10.1073/pnas.0811045106 19339495 PMC2664152

[46] Brown JR Goldblatt D Buddle J Morton L Thrasher AJ Diminished production of anti-inflammatory mediators during neutrophil apoptosis and macrophage phagocytosis in chronic granulomatous disease (CGD). J Leukoc Biol (2003) 73:591–9 10.1189/jlb.1202599 12714573

[47] Frasch SC Berry KZ Fernandez-Boyanapalli R Jin HS Leslie C Henson PM NADPH oxidase-dependent generation of lysophosphatidylserine enhances clearance of activated and dying neutrophils via G2A. J Biol Chem (2008) 283:33736–49 10.1074/jbc.M807047200 18824544 PMC2586279

[48] Sanmun D Witasp E Jitkaew S Tyurina YY Kagan VE Ahlin A Involvement of a functional NADPH oxidase in neutrophils and macrophages during programmed cell clearance: implications for chronic granulomatous disease. Am J Physiol Cell Physiol (2009) 297:C621–31 10.1152/ajpcell.00651.2008 19570889

[49] Min JK Kim YM Kim SW Kwon MC Kong YY Hwang IK TNF-related activation-induced cytokine enhances leukocyte adhesiveness: induction of ICAM-1 and VCAM-1 via TNF receptor-associated factor and protein kinase C-dependent NF-kappaB activation in endothelial cells. J Immunol (2005) 175:531–40 10.4049/jimmunol.175.1.531 15972689

[50] Lo SK Janakidevi K Lai L Malik AB Hydrogen peroxide-induced increase in endothelial adhesiveness is dependent on ICAM-1 activation. Am J Physiol (1993) 264:L406–12 7682787 10.1152/ajplung.1993.264.4.L406

[51] Chen XL Zhang Q Zhao R Ding X Tummala PE Medford RM Rac1 and superoxide are required for the expression of cell adhesion molecules induced by tumor necrosis factor-alpha in endothelial cells. J Pharmacol Exp Ther (2003) 305:573–80 10.1124/jpet.102.047894 12606638

[52] Kim SR Bae YH Bae SK Choi KS Yoon KH Koo TH Visfatin enhances ICAM-1 and VCAM-1 expression through ROS-dependent NF-kappaB activation in endothelial cells. Biochim Biophys Acta (2008) 1783:886–95 10.1016/j.bbamcr.2008.01.004 18241674

[53] Chen H Song YS Chan PH Inhibition of NADPH oxidase is neuroprotective after ischemia-reperfusion. J Cereb Blood Flow Metab (2009) 29:1262–72 10.1038/jcbfm.2009.47 19417757 PMC2733333

[54] Vendrov AE Hakim ZS Madamanchi NR Rojas M Madamanchi C Runge MS Atherosclerosis is attenuated by limiting superoxide generation in both macrophages and vessel wall cells. Arterioscler Thromb Vasc Biol (2007) 27:2714–21 10.1161/ATVBAHA.107.152629 17823367

[55] Martindale JL Holbrook NJ Cellular response to oxidative stress: signaling for suicide and survival. J Cell Physiol (2002) 192:1–15 10.1002/jcp.10119 12115731

[56] Balla G Jacob HS Balla J Rosenberg M Nath K Apple F Ferritin: a cytoprotective antioxidant strategem of endothelium. J Biol Chem (1992) 267:18148–53 1517245

[57] Fortes GB Alves LS de Oliveira R Dutra FF Rodrigues D Fernandez PL Heme induces programmed necrosis on macrophages through autocrine TNF and ROS production. Blood (2012) 119:2368–75 10.1182/blood-2011-08-375303 22262768 PMC3358230

