# Supplementary material for: Genetic Dissection of the Ity3 Locus Identifies a Role for Ncf2 Co-Expression Modules and Suggests Selp as a Candidate Gene Underlying the Ity3.2 Locus
Source: Front Immunol. 2014 Aug 12;5:375. doi: 10.3389/fimmu.2014.00375 (PMC4129629; doi:10.3389/fimmu.2014.00375)
Supplement: Supplementary file 1 [file Presentation1.ZIP › Supp figures.PDF]

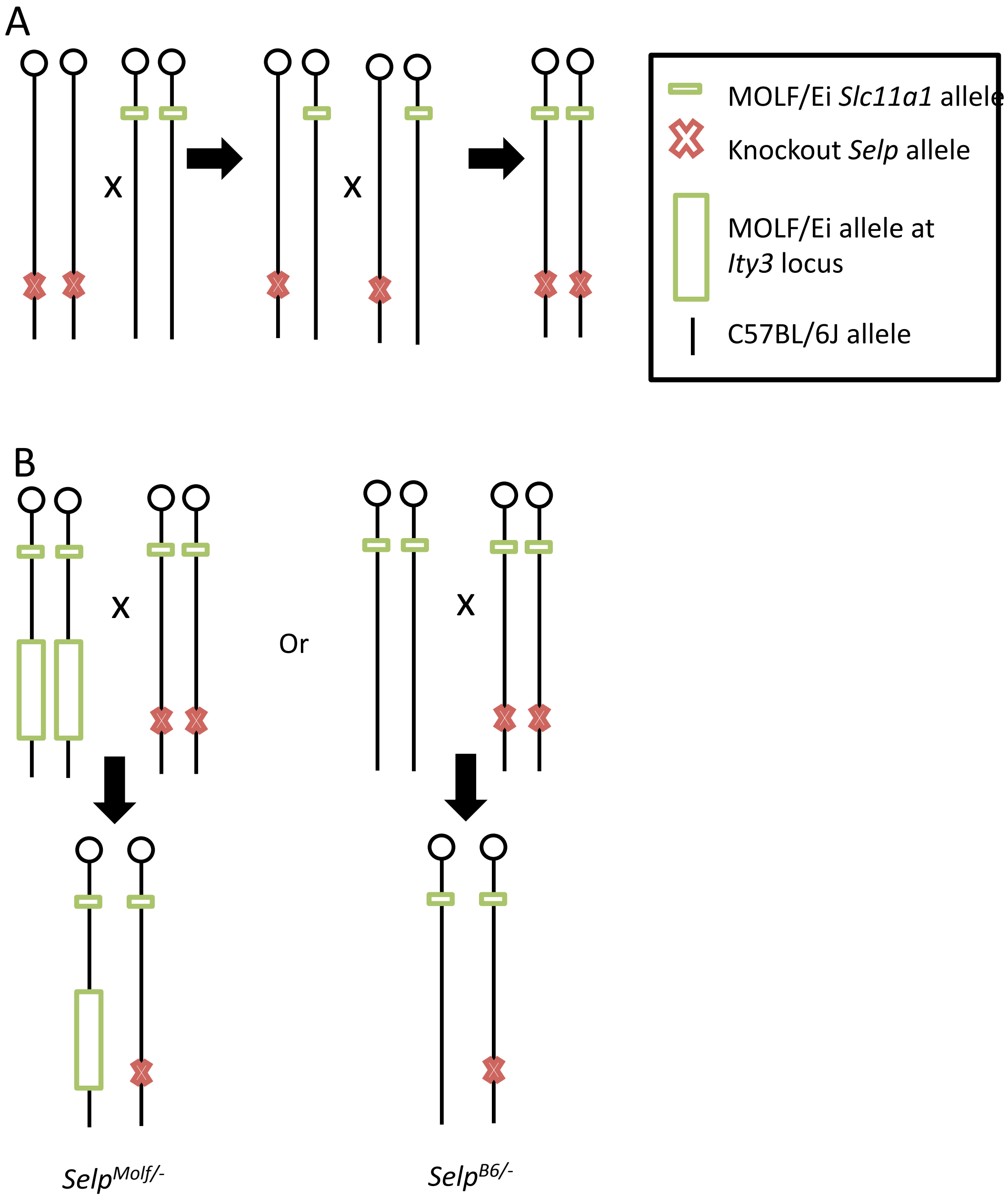

Supplemental Figure 1: Breeding scheme used to generate Selp-/-, Selp -/MOLF and Selp-/B6 mice. **(A)** Selp knockout mice (Selp-/-) were crossed with Ity mice and mice carrying the knockout allele at Selp and the MOLF/Ei allele at the Ity locus were inter-crossed to generate mice that are homozygous for the MOLF/Ei allele at the Ity locus and have the knockout allele at the Selp gene. **(B)** The homozygous Selp-/- mice were crossed with either the Ity or Ity3 mice to generate mice carrying one allele of the knockout Selp gene complemented by either the C57BL/6J allele (Selp-/B6) or the MOLF/Ei allele at the Selp gene (Selp-/MOLF).

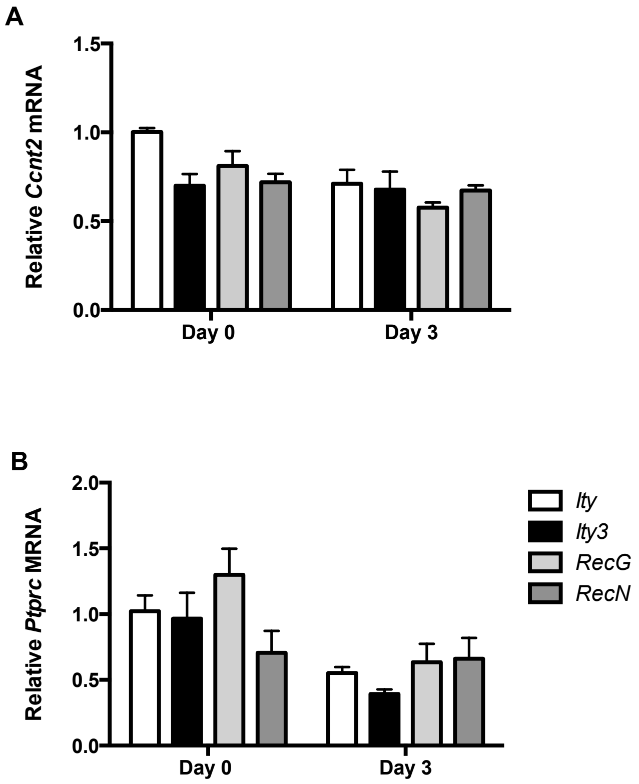

Supplemental Figure 2: Real-time PCR validation of two chromosome 1 genes identified using microarray analysis and showing lower expression levels in *Ity3* and *Ity3.RecG,* compared to *Ity* and *Ity3.RecN*. Relative expression ratios to the housekeeping gene, *Hprt*, were shown for the genes *Ccnt2* (A) and *Ptprc* (B). Primers specific to both C57BL/6J and MOLF/Ei sequences were selected. There were no major differences in gene expression among genotypes within each time point (2-way ANOVA) for both genes. These data are consistent with the fact that low levels of expression observed in microarray analysis could be consequence of poor hypridization of MOLF/Ei cDNA to the arrays.
